# Discovery of a New Cu‐Based Chalcogenide with High *zT* Near Room Temperature: Low‐Cost Alternative for the Bi_2_Te_3_‐Based Thermoelectrics

**DOI:** 10.1002/adma.202420556

**Published:** 2025-03-21

**Authors:** Oleksandr Cherniushok, Taras Parashchuk, G. Jeffrey Snyder, Krzysztof T. Wojciechowski

**Affiliations:** ^1^ Thermoelectric Research Laboratory, Department of Inorganic Chemistry Faculty of Materials Science and Ceramics AGH University of Krakow Mickiewicza Ave. 30 Krakow 30–059 Poland; ^2^ Department of Materials Science and Engineering Northwestern University Evanston IL 60208 USA

**Keywords:** copper chalcogenides, energy conversion efficiency, phase transitions, thermoelectric materials, ultralow thermal conductivity, waste heat recovery

## Abstract

Copper‐based chalcogenides are cost‐effective and environmentally friendly thermoelectric (TE) materials for waste heat recovery. Despite demonstrating excellent thermoelectric performance, binary Cu_2_ *X* (*X* = S, Se, and Te) chalcogenides undergo superionic phase transitions above room temperature, leading to microstructural evolution and unstable properties. In this work, a new γ‐phase of Cu_6_Te_3‐_ *_x_* S_1+_ *_x_* (0 < *x* ≤ 1) is discovered, a narrow‐bandgap semiconductor with outstanding thermoelectric performance and high stability. By substituting Te with S in metallic Cu_6_Te_3_S, the crystal symmetry is modified and structural phase transitions are eliminated. The γ‐phase exhibits a significantly higher Seebeck coefficient of up to 200 µVK^−1^ compared to 8.8 µVK^−1^ for Cu_6_Te_3_S at room temperature due to optimized carrier concentration and increased effective mass. Cu_6_Te_3‐_ *_x_* S_1+_ *_x_* materials also demonstrate ultralow thermal conductivity (≈0.25 Wm^−1^K^−1^), which, in concert with improved power factors, enables a high *zT* of ≈1.1 at a relatively low temperature of 500 K. Unlike most Cu‐based chalcogenides, the γ‐phase exhibits excellent transport property stability across multiple thermal cycles, making it a cost‐effective and eco‐friendly alternative to Bi_2_Te_3_‐based materials. The developed Cu_6_Te_3‐_ *_x_* S_1+_ *_x_* is a promising candidate for thermoelectric converters in waste heat recovery, and its potential can be further extended to cooling applications through carrier concentration tuning.

## Graphical abstract

This study reports the discovery of a stable γ‐phase in Cu_6_Te_3‐_ *_x_* S_1+_ *_x_*, achieving high thermoelectric performance with a Seebeck coefficient up to 200 µVK⁻¹ and ultralow thermal conductivity (≈0.25 Wm⁻¹K⁻¹). The material eliminates phase transitions, exhibits a *zT* of ≈1.1 at 500 K, and offers a cost‐effective, eco‐friendly alternative for waste heat recovery and cooling applications.

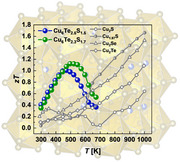

## 1 Introduction

The ever‐increasing demand for energy is driving society's need for new and more efficient energy sources.^[^ ^1^ ^]^ In this context, the thermoelectric (TE) phenomenon has received a new lease of life in the reality of global warming and the energy crisis facing humanity today.^[^ ^2^ ^]^ Thermoelectric materials can be used in renewable energy technologies to directly convert waste heat into electricity or to construct solid‐state heat pumps.^[^ ^3^, ^4^, ^5^ ^]^ The main limitation to the exploitation of TE‐based energy conversion technologies for broad industrial needs is the relatively low performance of the materials used in TE converters, which represents a significant challenge in this field.

The dimensionless figure of merit *zT* is commonly used to assess the energy conversion performance of TE materials:

(1) $$\;zT=\frac{S^{2}\sigma }{\kappa_{e}+\kappa_{L}}T$$

where *S* denotes the Seebeck coefficient, *σ* denotes the electrical conductivity, *T* denotes the absolute temperature, and *κ*_e_ and *κ*_L_ denote the electronic and lattice components of the thermal conductivity, respectively.^[^ ^6^ ^]^ A simple increase in the power factor *S* ^2^ *σ* is not necessarily enough to improve *zT*, because *S*, *σ*, and *κ*_e_ are related through the carrier concentration *n*.^[^ ^7^, ^8^, ^9^ ^]^ The lattice thermal conductivity *κ*_L_ is the only parameter that can be adjusted in a relatively independent way.^[^ ^10^, ^11^, ^12^ ^]^ Consequently, there has been a recent increase in research activity focused on the development of TE materials with low or even ultralow lattice thermal conductivity.^[^ ^10^, ^13^, ^14^, ^15^ ^]^ It is important to note, however, that the sole observation of low *κ*_L_ in TE materials does not necessarily indicate the high energy‐conversion performance of materials.^[^ ^16^, ^17^, ^18^ ^]^ A promising avenue for assessing the merit of TE materials is a recently proposed quality factor *B*, which is directly proportional to *μ*_w_/*κ*_L_ ratio, wherein *μ*_w_ represents the weighted mobility of the charge carriers.^[^ ^19^ ^]^ Consequently, the pursuit of superior TE materials should be guided by the objective of reaching high *μ*_w_/*κ*_L_, underscoring the significance of not only achieving ultralow lattice thermal conductivity but also of attaining high weighted mobility of charge carriers.^[^ ^20^, ^21^ ^]^

The extensive utilization of the most established TE materials, namely Bi_2_Te_3_, PbTe, GeTe, and TAGS, is constrained by two main factors: their toxic nature and the considerable variability in the cost of tellurium.^[^ ^11^, ^22^ ^]^ Consequently, the pursuit of novel earth‐abundant and tellurium‐free TE materials represents a significant challenge for researchers. In line with this concept, a considerable amount of attention from the materials engineering community is directed toward the advancement of copper‐based sulfides and selenides,^[^ ^23^, ^24^, ^25^, ^26^, ^27^ ^]^ which frequently exhibit highly promising TE properties. However, the majority of these compounds (e.g., Cu_2_ *X* and Cu‐based argyrodites) exhibit ionic conductivity, which limits their potential applicability due to electro‐thermal‐chemical stability challenges.^[^ ^28^, ^29^, ^30^, ^31^ ^]^ As an illustrative example, the optimized binary Cu_2_ *X* chalcogenides often exhibit markedly high thermoelectric performance, with a *zT* value of ≈2.0 or more.^[^ ^16^, ^32^, ^33^ ^]^ In particular, Cu_2_Se‐based materials exhibit high thermoelectric performance, such as a Cu_2_Se_0.92_S_0.08_ material with *zT* of 2.0 at 1000 K,^[^ ^34^ ^]^ Cu_2_Se‐BiCuSeO‐graphene composites with *zT* of 2.8 at 1000 K^[^ ^35^, ^36^ ^]^ and a Cu_1.99_Se–0.35 mol% AgSbF_6_ material with *zT* of 3.0 at 1050 K.^[^ ^32^ ^]^ However, due to their relatively wide band gap (more than 1 eV), the maximum performance is only achieved at very high temperatures, ≈1000 K where there is a greater challenge of stability.^[^ ^37^ ^]^ The tuning of bonding energy in Cu_2_Se_0.92_S_0.08_ material enhanced also the repeatability of thermoelectric properties,^[^ ^34^ ^]^ while compositing effects and ion confinement effectively mitigated ionic migration in Cu_2_Se‐BiCuSeO‐composites^[^ ^36^ ^]^ and AgSbF_6_ doped Cu_1.99_Se,^[^ ^32^ ^]^ respectively.

Stability concerns have thus far limited the implementation of most known Cu‐based chalcogenides in high‐temperature energy conversion devices. Second, there are no Cu‐based chalcogenides with good TE performance at low‐temperature range. Therefore, the design of new high‐performing Cu‐based chalcogenides for applications in the medium and low‐temperature range is of great importance. In this temperature region, only the Bi_2_Te_3_‐based materials with narrow band gaps and sufficient stability of properties are most commonly used for the fabrication of the TE converters.^[^ ^38^, ^39^ ^]^ Consequently, the search for narrow bandgap and stable Cu‐based chalcogenides as an alternative to bismuth telluride‐based materials appears to be a highly promising avenue of research.

Taking Cu‐based chalcogenides as a starting point in the search for new materials, our attention was drawn to the family of compounds with the β‐tungsten type of crystal structure (also known as A15‐type or Cr_3_Si‐type).^[^ ^40^, ^41^, ^42^, ^43^ ^]^ These phases can be described by the framework of closed‐packed icosahedra built up by the chalcogen atoms like in β‐W or Cr_3_Si, and Cu atoms fill part of tetrahedral voids in a disordered way.^[^ ^44^ ^]^ Regarding the transport properties, Giller et al. reported a Cu_6_Te_3_S compound as a metal‐like material with a very high electrical conductivity.^[^ ^44^ ^]^ Later Liu et al. reported relatively poor thermoelectric performance with *zT* < 0.2 at 600 K for this compound due to very high electrical conductivity and low Seebeck coefficient. However, through the substitution of Cu by Ag, the authors of this paper were able to tune the carrier concentration and achieve a reasonable *zT* of 0.7 at 600 K for Cu_4_Ag_2_Te_3_S.^[^ ^26^ ^]^ Similarly, Rabenbauer et al. reported that Cu_1.5_Se*_y_* Te_1−_ *_y_* (*y = *0.2−0.7) compounds exhibit characteristics of narrow‐bandgap semiconductors, featuring high electrical conductivity and a low Seebeck coefficient.^[^ ^45^ ^]^ Despite the very low thermal conductivity, due to low power factors, these materials show poor thermoelectric performance with *zT* of less than 0.2 at 573 K.

This paper presents the discovery of a new Cu‐based material with a β‐tungsten type of crystal structure, which exhibits high energy conversion performance at near‐room temperature. The chemical composition of this material, Cu_6_Te_3−_ *_x_* S_1+_ *_x_* (0 ≤ *x* ≤ 1), indicates the use of environmentally friendly and cost‐effective Cu and S, as well as a reduced Te content. The structural and thermoelectric properties of the discovered phase were investigated to ascertain its potential for utilization as a thermoelectric material in near‐room temperature regions. In addition to the excellent TE performance, it was found that the remarkable stability of the transport properties exhibited by the proposed Cu‐based chalcogenide renders it a genuine alternative to the conventional Bi_2_Te_3_‐based TE materials for thermoelectric converters.

## 2 Results and Discussion

### 2.1 Crystal Structure and Phase Analyses

**Figure 1:**
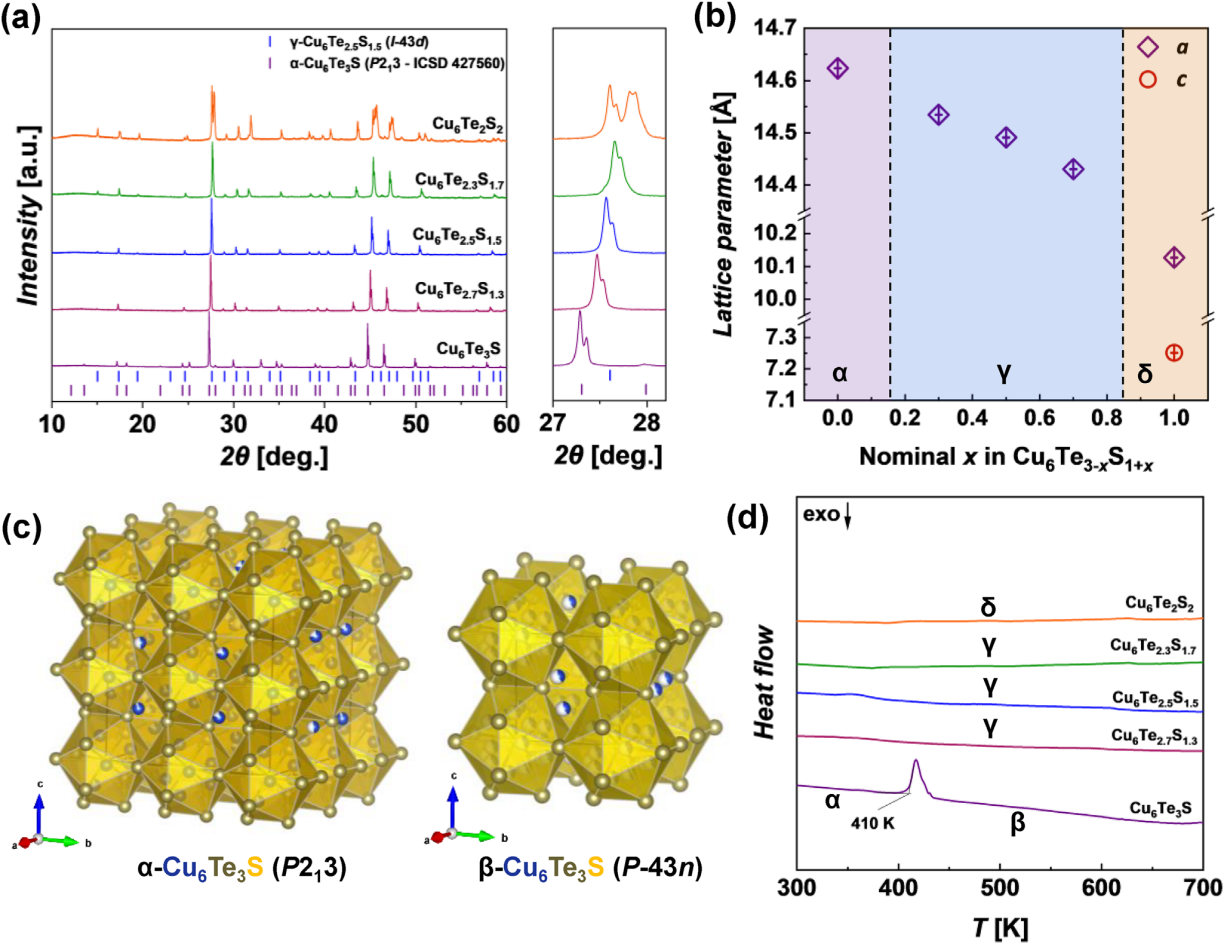
a) Powder XRD patterns (Cu *K*α radiation) and b) lattice parameters of the Cu_6_Te_3‐_ *_x_* S_1+_ *_x_* samples. c) Crystal structure representation of the α‐Cu_6_Te_3_S (space group *P*2_1_3) and β‐Cu_6_Te_3_S (space group *P*‐43*n*) polymorphic modifications. d) DSC curves of the Cu_6_Te_3‐_ *_x_* S_1+_ *_x_* samples with a marked temperature range of the existence of various polymorphic modifications.

Powder X‐ray diffraction (XRD) was used to analyze the phase composition of the synthesized Cu_6_Te_3−_ *_x_* S_1+_ *_x_* (0 ≤ *x* ≤ 1) samples and the results are presented in **Figures** **1**a and S1 (Supporting Information). The Cu_6_Te_3_S sample was indexed in the cubic *P*2_1_3 space group, which is associated with the low‐temperature α‐ polymorphic modification. This result is in agreement with Giller et al.,^[^ ^44^ ^]^ who reported that α‐Cu_6_Te_3_S crystallizes in the *P*2_1_3 space group (ICSD 427 560) at room temperature and exhibits a reversible phase transition at 404 K to another cubic β‐polymorph crystallizing in the *P‐*43*n* space group (ICSD 427 561) (Figure 1c). The crystal structure of Cu_6_Te_3_S is characterized by a 3D framework of icosahedra built up by Te atoms with S as the central atom, as it has also been described for the Cr_3_Si structure type. Giller et al. reported that in Cu_6_Te_3_S, weak bonding interactions are present between neighboring Te atoms (∼3.6 Å), as this interatomic distance is greater than a typical covalent bond (∼2.8 Å) but smaller than the van der Waals distance (∼4.12 Å).^[^ ^44^ ^]^ The Cu atoms occupy the part of the tetrahedral voids (which are the only type of voids) in the structure in a partially ordered way (α‐Cu_6_Te_3_S) or in a completely disordered way (β‐Cu_6_Te_3_S) (Figure 1c). As the sulfur content increases, the crystal structure of Cu_6_Te_3‐_ *_x_* S_1+_ *_x_* materials changes, and the corresponding powder patterns cannot be indexed in either the *P*2_1_3 space group or in the *P‐*43*n* space group. Instead, samples with *x* = 0.3, *x* = 0.5 and *x* = 0.7 can be indexed in the cubic *I*‐43*d* space group, as was reported by Goetzinger for the Cu_12_Te_5_S_3_ phase.^[^ ^46^ ^]^ This means, that in this compositional range, there is a new γ‐Cu_6_Te_3‐_ *_x_* S_1+_ *_x_* polymorph exists. Moreover, further addition of S in the sample with *x* = 1 causes structural distortion, which is seen in the reflection splitting. The XRD patterns of the mentioned sample can be indexed in tetragonal symmetry instead of cubic one. The crystal structure of the γ‐ and δ‐polymorphs has not yet been described in the literature and will be the topic of our next work.

As the sulfur content increases, the reflection positions consistently shift to higher 2*θ* values, indicating a reduction in lattice parameters (right panel in Figure 1a). Least squares refinement of the XRD reflection positions of the Cu_6_Te_3‐_ *_x_* S_1+_ *_x_* samples in the range of 10° ≤ 2*θ *≤ 100° was performed to estimate the lattice parameter changes (Figure 1b). Considering the fact that the atomic size of S is smaller than that of Te, the observed reduction of lattice parameters with S increasing in Cu_6_Te_3‐_ *_x_* S_1+_ *_x_* indicates the successful substitution of Te by S. But more importantly, it should also be mentioned that sulfur addition not only leads to the decrease of lattice parameters but also changes the crystal structure symmetry.

The thermal behavior of the investigated materials was studied using Differential Scanning Calorimetry (DSC) (Figure 1d). The obtained results show that α‐Cu_6_Te_3_S undergoes a structural phase transition to β‐Cu_6_Te_3_S at 410 K, which is in agreement with the literature data.^[^ ^44^ ^]^ Unexpectedly, the sulfur addition in Cu_6_Te_3‐_ *_x_* S_1+_ *_x_* not only changes the crystal structure symmetry but also completely eliminates the polymorphic phase transitions for the samples with *x* > 0 in the whole measured temperature range. This is a very important result in means of practical application of obtained materials for thermoelectric energy conversion because any structural transitions within the operating range cause degradation of the microstructure of the TE material.^[^ ^14^, ^47^ ^]^

**Figure 2:**
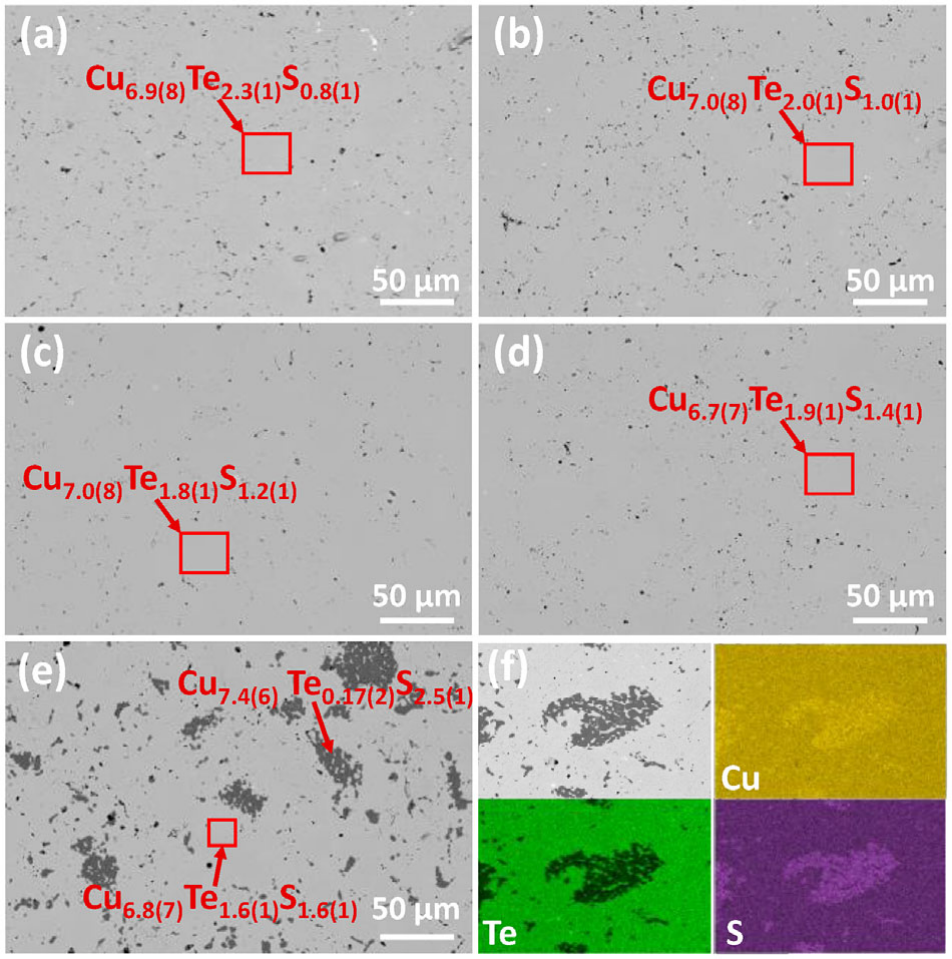
SEM backscattered electron images of the polished Cu_6_Te_3‐_ *_x_* S_1+_ *_x_* polycrystalline samples after LFA measurements: a) *x* = 0; b) *x*  = 0.3; c) *x* = 0.5; d) *x* = 0.7; e) *x* = 1, the chemical composition of phases determined using EDS analysis and marked in the SEM image. f) EDS elemental mapping images for the sample with *x* = 1.

The microstructural and chemical analysis of the samples after spark plasma sintering (SPS) treatment was analyzed by scanning electron microscopy (SEM) (**Figure** **2**). The microstructure images of the Cu_6_Te_3‐_ *_x_* S_1+_ *_x_* bulk samples indicate their predominantly single‐phase nature for most compositions, with low porosity, consistent with the high densification achieved (>97% of the theoretical density). The grain size remained below 50 µm after SPS sintering, with a small number of pores located at the grain boundaries. Energy‐dispersive X‐ray spectroscopy (EDS) point analysis confirmed good agreement between the measured and nominal chemical compositions, although a slightly higher copper content was observed. The discrepancies in chemical composition arise from the accuracy limitations of the EDS analysis, where the error for copper was ≈10% for each sample. Taking this error into account and applying the 3σ‐rule, we can assert that our EDS compositions are in agreement with the nominal values, considering the inherent accuracy constraints of EDS chemical analysis. The backscattered electron image of the Cu_6_Te_3‐_ *_x_* S_1+_ *_x_* sample with *x* = 1 revealed the presence of darker regions, which, as determined by EDS point analysis, are richer in sulfur and copper (Figure 2e). The EDS elemental mapping further confirmed this observation (Figure 2f). This composition (*x* = 1) crosses the solubility limit of sulfur in Cu_6_Te_3‐_ *_x_* S_1+_ *_x_*, leading to the formation of precipitates appearing either as isolated grains of several microns in size or as agglomerates reaching up to 50 microns. In contrast, EDS analysis of the sample with *x* = 0.7 confirmed its single‐phase nature, with a homogeneous elemental distribution (Figure S2, Supporting Information).

**Figure 3:**
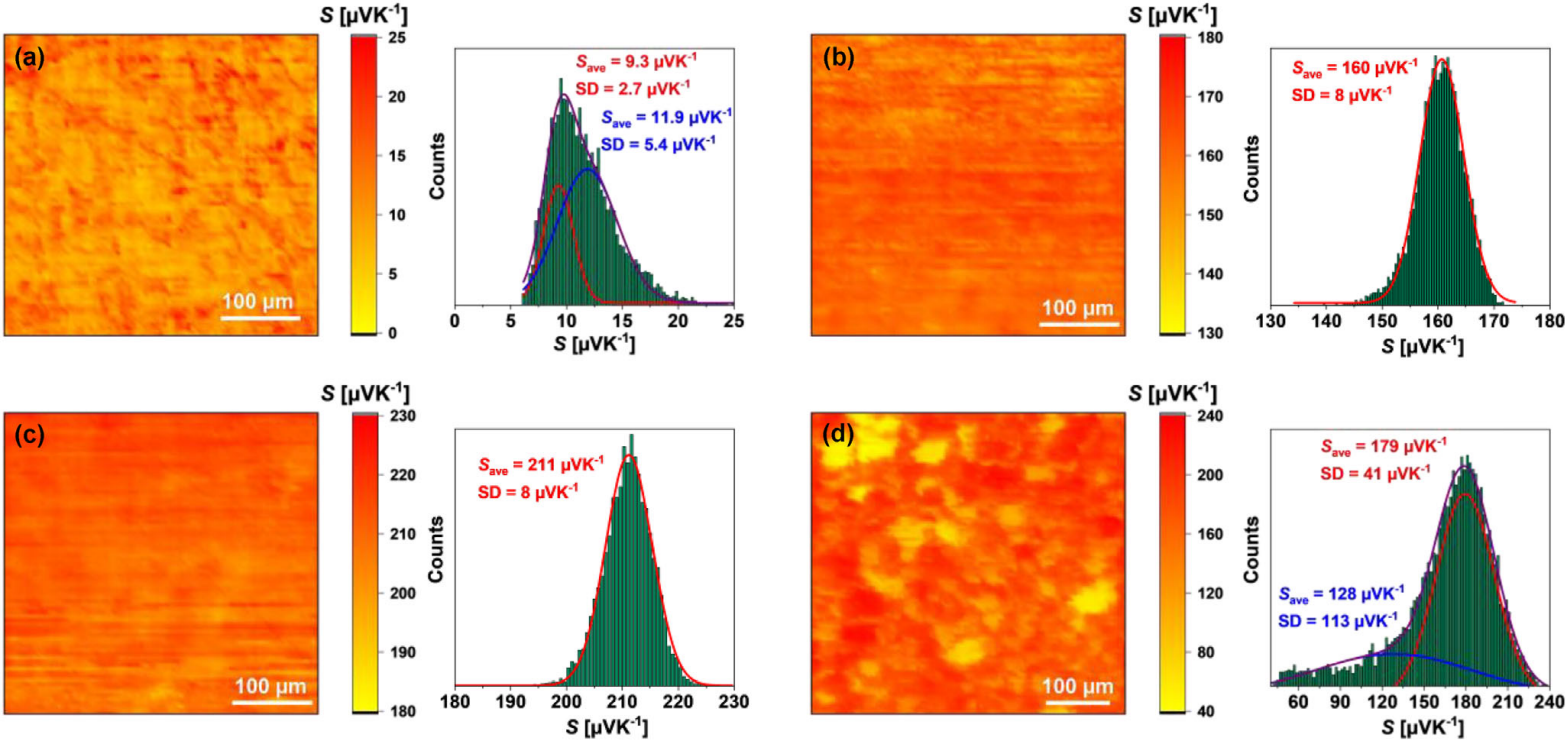
Seebeck coefficient SThM maps scanned on a polished surface of the Cu_6_Te_3‐_ *_x_* S_1+_ *_x_* polycrystalline samples after LFA measurements: a) *x* = 0; b) *x* = 0.5; c) *x* = 0.7; d) *x* = 1.

In order to verify the uniformity of the Cu_6_Te_3‐_ *_x_* S_1+_ *_x_* specimens under investigation, we have carried out Scanning Thermoelectric Microprobe (SThM) scans (**Figure** **3**a–d; Figure S3, Supporting Information). Each map in Figure 3 represents the plot of 10 000 independent Seebeck coefficient (*S*) measurements on the polished surface of the specimen. These datasets were analyzed by plotting the histograms of the Seebeck coefficients and fitting them with an unimodal or bimodal Gaussian distribution function. The average Seebeck coefficient values were in good agreement with those measured at room temperature (using the NETZSCH SBA 458 *Nemesis* apparatus), as presented in the following section. Samples with *x* = 0.3, *x* = 0.5, and *x* = 0.7 (Figure S3, Supporting Information and Figure 3b,c) which represent γ‐polymorph, show a high spacial homogeneity of the Seebeck coefficient and were well fitted with the unimodal Gaussian distribution function. Nonetheless, a sample with *x* = 0, could not be fitted well with unimodal distribution, instead bimodal one had to be used. This can be related to the structural transition this material undergoes, which can cause grain boundary degradation and influence the Seebeck coefficient. As observed in the SEM backscattered images, the sample with *x* = 1 exhibited the presence of secondary phases. These phases are expected to strongly influence the Seebeck coefficient, and the SThM technique serves as a valuable tool to investigate such effects. In particular, this unique equipment allows to measure the local Seebeck coefficient over the polished surface of samples, if the materials exhibit different phases even with very similar chemical compositions, the Seebeck coefficient will be different due to different carrier concentrations. Figure 3d presents the SThM measurement results, where brighter regions correspond to areas with lower Seebeck coefficient values. What is also very interesting, a direct correlation can be observed between the shape and size of the brighter regions in the SThM maps and the inclusions detected in SEM images. These secondary phase inclusions arise due to the exceeding of sulfur solubility in the Cu_6_Te_3‐_ *_x_* S_1+_ *_x_* phase, leading to the formation of precipitates. Based on EDS analysis, we hypothesize that these inclusions correspond to the secondary phase with a higher carrier concentration, due to a lower Seebeck coefficient. The increase in carrier concentration in the *x* = 1 sample in comparison to *x* = 0.7 was also observed on Hall measurements described in the next section. This is consistent with previous findings in diamond‐like Cu_2_CoSnS_4‐_ *_x_* Se*_x_* materials,^[^ ^13^, ^48^ ^]^ where secondary phases were found to influence charge carrier transport. In that case, charge carriers from the secondary phase could be thermally activated at elevated temperatures, contributing to enhanced electronic transport. Probably such a way of thinking could be extended to the materials investigated in this work, and we will have to keep this result in mind later when analyzing the temperature‐dependent electronic transport.

### 2.2 Transport Properties

The Seebeck coefficients of the Cu_6_Te_3‐_ *_x_* S_1+_ *_x_* samples at 298 K indicate an ascending tendency with increasing sulfur content (**Table** **1**). The discrepancy from the overall tendency for the sample with *x*  =  1 can be connected with the presence of a secondary phase which was determined by the SEM/EDS and SThM analyses. The Seebeck coefficient of the Cu_6_Te_3_S material shows a very low value of 8.8 µVK^−1^ due to a high carrier concentration of 1.39 × 10^21^ cm^−3^. Such a low value of the Seebeck coefficient and high carrier concentration are indicators of rather poor thermoelectric performance for this material. The situation has been significantly different for the case of the S‐substituted samples. The materials with *x*  =  0.3–0.7 show an order of magnitude or more increase in the Seebeck coefficient (from 8.8 µVK^−1^ for Cu_6_Te_3_S up to 199.7 µVK^−1^ for Cu_6_Te_2.3_S_1.7_) suggesting the significant but still not clear role of the S/Te substitution in optimizing the carrier concentration toward high TE performance. But what is even more interesting, the density of electronic state (DOS) effective mass (m*) for these samples is very different (details of DOS *m* ^*^ calculations are provided in the Supporting information and in our prior papers).^[^ ^9^, ^49^ ^]^ Particularly, the m* values for the S‐substituted materials are ≈1*m*_e_, while the effective mass for Cu_6_Te_3_S is only 0.19*m*_e_. This indicates also that except for the large changes in the carrier concentration *n*_H_, the substitution of Te by S atoms is also reflected in the changes in the band structure, as is expected due to the different crystal structures. Typically, an increase in effective mass m* leads to a decrease of carrier mobility.^[^ ^50^ ^]^ However, in the case of the *γ*‐Cu_6_Te_3‐_ *_x_* S_1+_ *_x_* samples, we observe a more than fivefold increase in DOS effective mass while maintaining or even significantly enhancing Hall carrier mobility (Figure S4, Supporting Information). Based on the current understanding of these novel materials, the most probable explanation for this phenomenon is the higher band degeneracy *N_V_* in the *γ*‐Cu_6_Te_3‐_ *_x_* S_1+_ *_x_* in comparison to α‐Cu_6_Te_3_S. A similar trend of m* has been reported for Cu_7_P(S*_x_* Se_1−_ *_x_*)_6_ argyrodites, where sulfur substitution in the high‐symmetry *γ*‐phase results in a multivalley band structure.^[^ ^15^ ^]^ In this case, the combination of a large m* and enhanced carrier mobility *µ*_H_ suggests an improvement in the electronic transport properties of *γ*‐Cu_6_Te_3−_ *_x_* S_1+_ *_x_* materials.

**Table 1:** Electrical and thermoelectric properties of Cu_6_Te_3−_ *_x_* S_1+_ *_x_* samples at 298 K, including the Seebeck coefficient (*S*), electrical conductivity (*σ*), thermal conductivity (*κ*), Hall concentration (*n*_H_), Hall carrier mobility (*µ*_H_), and density of states effective mass (*m* ^⁎^).

Cu_6_Te_3−_ *_x_* S_1+_ *_x_*	*S*, µV K^−1^	*σ*, S cm^−1^	*κ*, W m^−1^ K^−1^	*n*_H,_ cm^−3^	*µ*_H_, cm^2^ V^−1^ s^−1^	m^*^/m_e_
*x = *0	8.8	3611.7	2.21	1.39 × 10^21^	16.3	0.19
*x = *0.3	81.8	441.0	0.42	2.00 × 10^20^	13.8	1.38
*x = *0.5	156.4	146.0	0.26	3.12 × 10^19^	29.2	1.02
*x = *0.7	199.7	71.7	0.24	1.57 × 10^19^	28.5	0.99
*x = *1	146.8	84.9	0.28	3.73 × 10^19^	14.2	1.07

Consistent with the evolution of the Seebeck coefficients, the electrical conductivity *σ* decreases significantly in the series with the rise of *S* content, and again the sample with *x* = 1 deviates from the trend due to the presence of more conductive precipitates. What is very interesting is the fact that sulfur alloying of the Cu_6_Te_3−_ *_x_* S_1+_ *_x_* decreases carrier concentration *n*_H_ by two orders of magnitude in comparison to metallic Cu_6_Te_3_S. This is a rather unexpected phenomenon considering the isoelectronic substitution of Te by S and is most probably related to the strengthening of chemical bonds which limits the formation of Cu vacancies that generate positively charged carriers.

The other “black horse” of these materials is that the significant increase in Hall carrier concentration *n*_H_ causes only moderate changes in carrier mobility. The nature of such an observation is not clear and can be connected with the combined effect of several factors caused by the S/Te substitution, e.g. increase in the band gap, Cu vacancies formation, and modification of the band structure. We should also mention that the recorded carrier mobility *µ*_H_ is in the range of 16.3–29.2 cm^2^ V^−1^ s^−1^ and is significantly higher in comparison to state‐of‐the‐art Cu‐based “liquid‐like” materials.^[^ ^15^, ^16^, ^51^ ^]^

The thermal conductivity *κ* of the investigated Cu_6_Te_3−_ *_x_* S_1+_ *_x_* materials is also shown in Table 1. The values of *κ* for the Cu_6_Te_3_S equals 2.21 W m^−1^ K^−1^ at 298 K which is consistent with the values reported by Liu et al.^[^ ^26^ ^]^ The addition of S instead of Te in Cu_6_Te_3−_ *_x_* S_1+_ *_x_* drastically reduces the thermal conductivity from 2.21 W m^−1^ K^−1^ to 0.24 W m^−1^ K^−1^ at 298 K. Of course, such a change can be caused by the heat transported by the charge carriers due to large differences in electrical conductivity. To analyze this issue, we first calculated the lattice thermal conductivity and then performed the deep analysis of the *κ*_L_ values for the investigated materials. The results of this analysis are shown in the following section.

**Figure 4:**
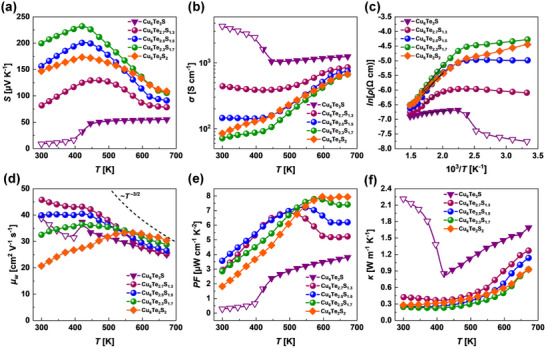
Temperature‐dependent (a) Seebeck coefficient, b) electrical conductivity, c) Arrhenius plot of electrical resistivity, d) weighted mobility, e) power factor, and f) thermal conductivity of Cu_6_Te_3−_ *_x_* S_1+_ *_x_* materials. For the Cu_6_Te_3_S sample, open symbols represent the temperature range where the α‐modification exists, while filled symbols indicate the range of β‐modification.

The temperature‐dependent Seebeck coefficients for the Cu_6_Te_3−_ *_x_* S_1+_ *_x_* materials are shown in **Figure** **4**a. All examined samples exhibit positive Seebeck coefficient values across the entire temperature range, confirming that holes serve as the primary charge carriers. The Seebeck coefficient (*S*) of initial Cu_6_Te_3_S shows the lowest temperature dependence *S*(*T*), while the sulfur substitution significantly increases the Seebeck coefficient over the entire investigated temperature range. The β‐Cu_6_Te_3_S phase, which occurs after the structural transformation at 410 K, possesses a significantly higher Seebeck coefficient in comparison to α‐Cu_6_Te_3_S, which may also indicate changes in the band structure between these two polymorphs. The other possible reason that could be involved here for the explanation of the change of the Seebeck coefficient, is the enhancement of the grain boundary effect due to the microstructure degradation after the phase transition. Such an effect was recently discovered in the case of Cu‐based argyrodites.^[^ ^47^ ^]^ A bell‐shaped temperature trend of the Seebeck coefficient, indicating minority carrier activation, is observed in most samples. With the S/Te substitution in the Cu_6_Te_3−_ *_x_* S_1+_ *_x_* materials the *S*(*T*) maximum shifts to the lower temperatures due to the decrease of the carrier concentration. The observation of *S*(*T*) maximum at the investigated temperature range made it possible to employ the Goldsmid‐Sharp equation (*E_g_* =  2*e* *S_max_T_max_*) for the estimation of the values of the band gap (Figure S5, Supporting Information). The results of the calculations show that the sulfur alloying increases the band gap of Cu_6_Te_3−_ *_x_* S_1+_ *_x_* materials from 0.07 eV for the sample with *x* = 0, to ≈0.2 eV for the sample with *x* = 0.7. The band gap for the materials with *x* > 0 seems to be more optimal considering the known optimization criteria for TE materials proposed by A. Ioffe *E*_g_ ≈8*k*_B_ *T*, where *k*_B_ stands for the Boltzmann constant and *T* for the temperature.

The electrical conductivity for the Cu_6_Te_3−_ *_x_* S_1+_ *_x_* materials over the entire temperature range from 298 to 673 K is shown in Figure 4b. Most of the samples show metal‐semiconductor transition which is shifting to lower temperatures due to the opening of band gap and decreasing of carrier concentration with sulfur substitution. In the high‐temperature region, all samples show intrinsic semiconducting behavior. Therefore, from the Arrhenius plot of electrical resistivity, we were able to estimate the conduction activation energies (*E_a_* =   − A *k_B_*) (Figure 4c) which are in good agreement with the band gaps previously obtained from the temperature trends of Seebeck coefficients (Figure S5, Supporting Information). In agreement with the *S*(*T*) dependencies, the electrical conductivity of the pristine Cu_6_Te_3_S shows the highest trend due to a significantly larger carrier concentration *n*_H_. But what is even more interesting, α‐Cu_6_Te_3_S shows metal‐like behavior while β‐Cu_6_Te_3_S shows intrinsic semiconducting behavior. Since the intrinsic semiconductor must be valence‐balanced, the charge balance in Cu_6_Te_3_S can be realized by the weak Te‐Te bonding interactions, so the negative charge might not be as extreme as 8^−^. This hypothesis seems realistic as Giller et al. reported that the presence of Cu^2+^ is unlikely based on XPS measurements.^[^ ^44^ ^]^

For evaluating the feasibility of the designed materials and for testing the TE performance change due to the S/Te substitution, the weighted mobility was calculated for the studied Cu_6_Te_3−_ *_x_* S_1+_ *_x_* materials (Figure 4d).^[^ ^21^ ^]^ Notably, in the high‐temperature region, we observed the growth of the weighted mobility in the series with increasing sulfur content, and the temperature trend also roughly follows ≈*T* ^−3/2^, suggesting that charge carriers are mostly scattered at acoustic or/and optical phonons. Conversely, in the low‐temperature range with increasing sulfur content, we observe a suppression of the weighted mobility for most of the samples, and even an increasing temperature trend for the samples with the highest S content, which may be an indicator of grain boundary resistance. The increasing trend of *µ*_W_(*T*) observed in Cu_6_Te_2_S_2_ is most likely attributed to charge carrier scattering occurring at both grain boundaries and impurity phases.^[^ ^52^ ^]^ The presence of grain boundaries can introduce potential barriers that hinder carrier transport, while secondary phases may further contribute to scattering by creating additional interfaces within the material. Interestingly, β‐Cu_6_Te_3_S also shows a higher trend of weighted mobility compared to α‐Cu_6_Te_3_S, which can confirm the better thermoelectric performance of this polymorph.

The calculated power factors *PFs* (*PF = S^2^σ*) are shown in Figure 4e. The Cu_6_Te_3_S sample has the poorest power factor throughout the entire temperature range, mainly due to the very low values of Seebeck coefficients. For the other samples, at elevated temperatures, increasing the sulfur content in Cu_6_Te_3−_ *_x_* S_1+_ *_x_* increases the power factor and shifts its maximum to higher temperatures. Such *PF* behavior is rather unexpected and can be explained probably due to the increase in the band gap and change in the carrier scattering mechanism.

The total thermal conductivity as a function of temperature for the Cu_6_Te_3−_ *_x_* S_1+_ *_x_* samples is shown in Figure 4f. The temperature dependence of electrical conductivity is well reflected in the temperature trends of *κ* for all samples. The Cu_6_Te_3_S material has the highest values of thermal conductivity mainly due to the highest electronic contribution to the total *κ* caused by its high electrical conductivity. The remaining samples which have significantly lower electrical conductivity *σ*, exhibit extremely low thermal conductivity at room temperature and start to increase after 423 K most probably due to the bipolar conduction.

To test the above hypothesis, the lattice thermal conductivity *κ*_L_ was estimated by subtraction of the electronic contribution *κ*_e_ from the total *κ*. We keep in mind that above 423 K the bipolar contribution *κ*_b_ to the total thermal conductivity becomes significant, therefore at the elevated temperature we focused not only on *κ*_L_, but rather *κ*_L_+ *κ*_b_ after subtracting the electronic part (Figure S6, Supporting Information). The calculations were performed using the Kane band (SKB) model considering the acoustic phonon scattering as the dominant scattering mechanism. The DOS effective masses required for the calculations were obtained from measured Hall carrier concentrations and Seebeck coefficients. The details of the calculations can be found in the Supporting Information. We first calculated electronic thermal conductivity using the equation *κ*_e_ = *σTL*_SKB_. For Cu_6_Te_3−_ *_x_* S_1+_ *_x_* samples with *x* > 0, this approach resulted in reasonable values of both *κ*_e_ and *κ*_L_. Therefore, we expected that this model would also work for the sample with *x* = 0. Nevertheless, for the Cu_6_Te_3_S sample calculated *L*_SKB_ values (Figure S6b, Supporting Information) led to unrealistically high values of *κ*_e_ which even exceed *κ*_total_ values (Figure S6a, Supporting Information). Authors of the paper^[^ ^26^ ^]^ also faced this problem estimating the lattice thermal conductivity of the Cu_6_Te_3_S chalcogenide using the single parabolic band (SPB) model approximated as $L=1.5+exp[-\frac{\left|S\right|}{116}]$. Therefore, despite the metallic behavior and very low values of the Seebeck coefficient, Cu_6_Te_3_S should be rather treated as a low Lorenz‐number material. Moreover, the mentioned conclusion is in line with the observation that the rest of the Cu_6_Te_3−_ *_x_* S_1+_ *_x_* samples are characterized by values of *L* well below the nondegenerate limit of *L* = 1.5  ×  10^−8^ W Ω K^−2^. Thus, for this sample, we estimated Lorenz numbers from Wiedemann–Franz law (*κ*_total_ = *κ*_L_ + *σTL*) by plotting *κ*_total_ versus *σ* at a fixed temperature for single‐phase Cu_6_Te_3−_ *_x_* S_1+_ *_x_* samples. As a result, we have got linear *κ*_total_ versus *σ* dependence at each fixed temperature, where intercept describes *κ*_L_ while a slope is equal to *L*_isothermal_  × *T*, and defined *L*_isothermal_ at each given temperature (Figure S6c,d, Supporting Information). The results of the calculation of *κ*_e_ using a classical and *L*_isothermal_ for Cu_6_Te_3_S sample are shown in Figure S6a,b (Supporting Information).

Similar behavior of Lorenz number was observed also for CaAgSb_1−_ *_x_* Bi*_x_* Zintl phases with complex crystal structure, which also aroused issue with “negative” lattice thermal conductivity.^[^ ^53^ ^]^ A low Lorenz number in Cu_6_Te_3−_ *_x_* S_1+_ *_x_* can be an indicator of decoupling lattice and electronic thermal conductivities, leading to greater flexibility for tailoring thermoelectric properties.

Figure S6a (Supporting Information) presents the lattice thermal conductivity of Cu_6_Te_3−_ *_x_* S_1+_ *_x_* samples as a function of temperature. All the investigated materials are characterized by extremely low *κ*_L_, within 0.2–0.25 Wm^−1^K^−1^. These exceptionally low *κ*_L_ values are considerably lower than those of state‐of‐the‐art thermoelectric materials,^[^ ^16^, ^17^, ^54^, ^55^ ^]^ and even lower compared to the liquid‐like Cu_2_(S,Se,Te) chalcogenides.^[^ ^16^, ^25^ ^]^ In turn, the thermal conductivity of Cu_6_Te_3−_ *_x_* S_1+_ *_x_* materials is comparable to that of argyrodites.^[^ ^1^ ^]^ This phenomenon is described in the literature as the migration or “liquid‐like behavior” of Cu⁺ ions within the rigid framework created by the chalcogen atoms. However, in the case of the investigated Cu_6_Te_3−_ *_x_* S_1+_ *_x_* chalcogenides, Cu ion migration is less probable, as these materials operate at relatively low temperatures compared to the high‐temperature liquid‐like Cu_2_(S,Se,Te) chalcogenides. The very recent work of Ghata et al. contradicts the “liquid‐like” paradigm and proposes the alternative strategy that the ultralow lattice thermal conductivity in argyrodites is caused by the complexity of the structure, the variation in bond strength and the high degree of lattice anharmonicity.^[^ ^56^ ^]^ In our opinion, such an explanation can also be applied here as well. In the work of Liu et al.,^[^ ^26^ ^]^ the low thermal conductivity of Cu_6_Te_3_S was attributed to the presence of various types of interatomic interactions with generally weak chemical bonding. This, combined with the large unit cell, results in a low cut‐off frequency of acoustic phonons and high lattice anharmonicity, which significantly suppresses heat transport. As a good way to shed some light on the problem of *κ*_L_ in the investigated new Cu‐based chalcogenides, we found very useful the ultrasonic measurements, which characterize only the heat transport carried by phonons and are not affected by electronic and bipolar heat transport.

**Table** **2** presents the results of ultrasonic measurements conducted on the analyzed samples. Interestingly, the speed of sound of the Cu_6_Te_3_S sample is considerably higher than for the rest of the samples with higher sulfur content. Such an effect is surprising because the materials with light elements usually have a higher speed of sound propagation than the heavy ones.^[^ ^13^, ^14^ ^]^ For example, the average speed of sound of Cu_2_Te is 1833 m s^−1^,^[^ ^57^ ^]^ whereas Cu_2_S shows a significantly larger value of 1991 m s^−1^.^[^ ^58^ ^]^ The lower speed of sound for the Cu_6_Te_3−_ *_x_* S_1+_ *_x_* samples with *x* > 0 in comparison to binary Cu_2_S and Cu_2_Te can suggest softer chemical bonding in the investigated materials. This hypothesis could be supported by the reduced Young's modulus *E* in the Cu_6_Te_3−_ *_x_* S_1+_ *_x_* series (Table 2). The increase in the Grüneisen parameter *γ* in the series indicates the increase in the anharmonicity of the lattice vibrations, which can originate from the electron lone‐pairs on the Te atoms, as recently reported by Liu et al.^[^ ^26^ ^]^ in Cu_6_Te_3_S.

**Table 2:** The longitudinal *ν_l_*, transverse *ν_t,_* and average sound velocity *ν_m_*, the Debye temperatures Θ*_D_*, Bulk modulus *B*, Young modulus *E*, the Poisson ratio ν, Grüneisen parameter γ, phonon mean free path *l_ph_*, and the minimum thermal conductivity *κ_glass_* and *κ_diff_* for Cu_6_Te_3−_ *_x_* S_1+_ *_x_* samples.

Cu_6_Te_3−_ *_x_* S_1+_ *_x_*	*v_l_*, m s^−1^	*v_t_*, m s^−1^	*v_m_*, m s^−1^	Θ*_D_*, K	*B*, GPa	*E*, GPa	ν	*γ*	*l_ph_*, Å	*κ_glass_*, W m^−1^ K^−1^	*κ_diff_*, W m^−1^ K^−1^
*x = *0	3409	1754	1964	213.3	50.2	54.2	0.32	1.90	2.00	0.530	0.334
*x = *0.3	3206	1592	1787	198.7	45.8	45.0	0.34	2.02	2.11	0.496	0.312
*x = *0.5	3257	1608	1805	200.6	46.3	44.7	0.34	2.04	1.82	0.505	0.318
*x = *0.7	3207	1565	1758	196.9	45.3	42.4	0.34	2.08	1.91	0.499	0.314
*x = *1	3250	1556	1750	197.4	46.4	41.4	0.35	2.14	2.09	0.504	0.318

In the Cu_6_Te_3_S structure, Te and S have separate crystallographic positions like Cr and Si in the Cr_3_Si crystal structure prototype. However, in the Cu_6_Te_3−_ *_x_* S_1+_ *_x_* samples with *x* > 0, a mixed occupancy of Te/S is expected, forming a disorder chalcogen sublattice as in the β‐tungsten structure prototype. Such a disorder leads to the strengthening of phonon scattering and can explain the decrease in the speed of sound and thermal conductivity for samples with *x* > 0 compared to Cu_6_Te_3_S. Intriguingly, the lattice thermal conductivity of most of the Cu_6_Te_3−_ *_x_* S_1+_ *_x_* samples falls below the minimum thermal conductivity *κ*_diff_ values. A similar phenomenon was also observed in Cu‐based argyrodites and some other materials.^[^ ^14^, ^15^, ^59^ ^]^ This behavior may be attributed to the suppression of certain shear modes that do not propagate through the lattice, as indicated by the large difference between the measured in this work longitudinal and transverse speed of sound in Cu_6_Te_3−_ *_x_* S_1+_ *_x_* materials. Additionally, the large unit cells in these materials accommodate diverse cation‐anion interactions, leading to strong bonding inhomogeneity, which may also contribute to the breakdown of the *κ*_diff_ limit, as seen in NaAgGa_6_Te_10_ chalcogenide.^[^ ^10^ ^]^ Nevertheless, further development of heat transport models is needed to fully explain the ultralow lattice thermal conductivity observed in Cu_6_Te_3−_ *_x_* S_1+_ *_x_* chalcogenides.

**Figure 5:**
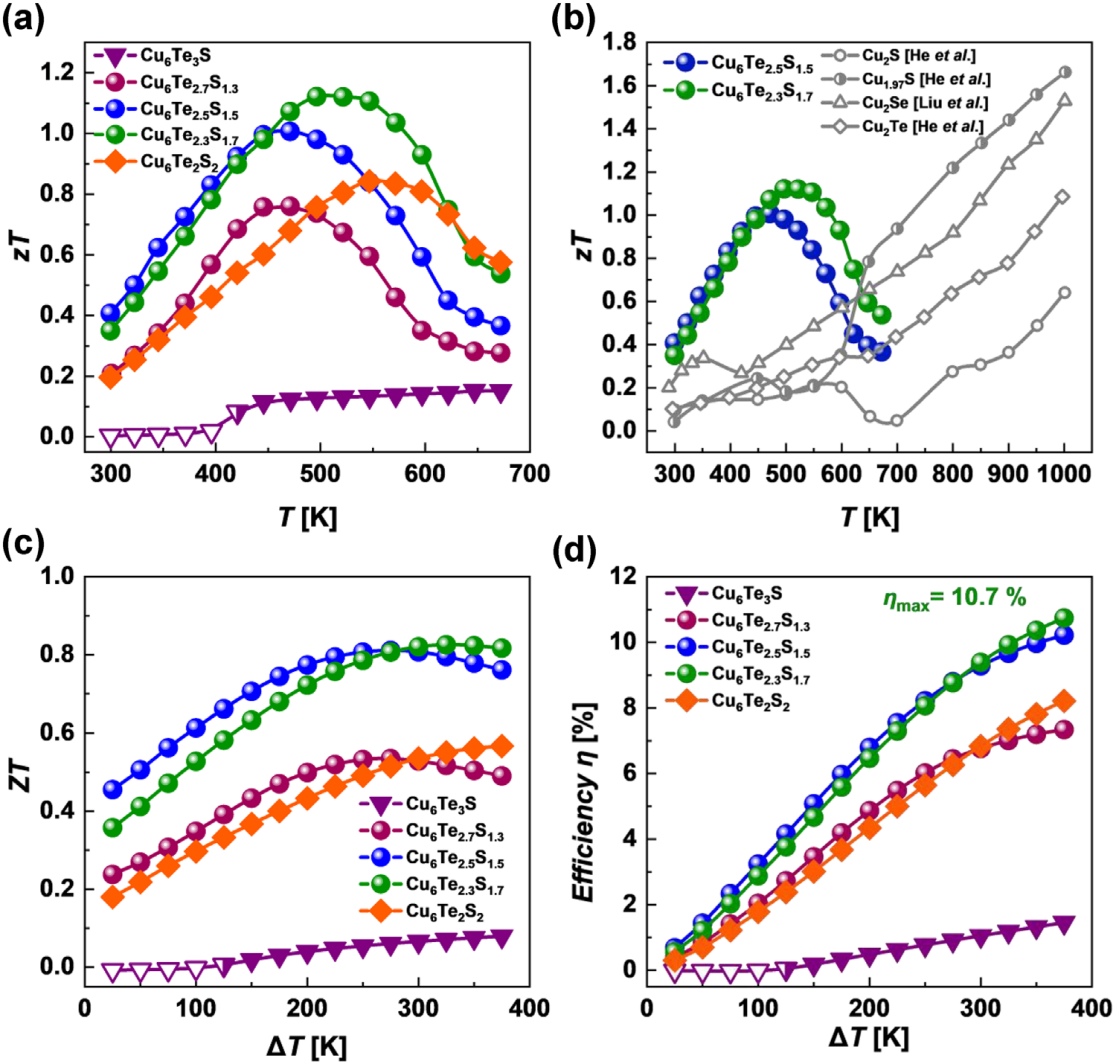
a) Dimensionless thermoelectric figure of merit *zT* for the Cu_6_Te_3−_ *_x_* S_1+_ *_x_* materials as a function of temperature and b) comparison of the achieved *zT* with the values given in the literature for undoped binary copper chalcogenides.^[^ ^63^, ^64^ ^]^ c) Device *ZT* and d) estimated energy conversion efficiency as a function of temperature gradient Δ*T* (Δ*T*  =  *T*_h_‐*T*_c_, where *T*_h_ and *T*_c_ (*T*_c = _298 K) are the hot side and cold side temperatures, respectively) for Cu_6_Te_3−_ *_x_* S_1+_ *_x_* materials.

The dimensionless thermoelectric figure of merit *zT* parameters were determined in order to assess the energy conversion performance of the designed Cu_6_Te_3−_ *_x_* S_1+_ *_x_* materials (**Figure** **5**a,b). Due to the lowest power factor in the series and high thermal conductivity, metallic Cu_6_Te_3_S shows a poor *zT* of 0.15 at 673 K. The synergistic effect of improved power factors and greatly reduced thermal conductivity (Figure 4e,f) results in a remarkable enhancement of the *zT* parameters of Cu_6_Te_3−_ *_x_* S_1+_ *_x_* materials with *x* > 0 (Figure 5a). Together with the above‐mentioned opening of the band gap in Cu_6_Te_3−_ *_x_* S_1+_ *_x_* materials, the maximum *zT* value shifts to the higher temperature region which can be useful for the development of functionally graded thermoelectric legs.^[^ ^60^, ^61^, ^62^ ^]^ Particularly, the *zT* parameter reaches a high value of 1.12 for the material with *x* = 0.7 and, what is even more important, at a relatively low temperature of 498 K.

This work reveals that sulfur substitution is an even more effective strategy for the enhancement of TE performance of Cu_6_Te_3_S than silver alloying, for which *zT = *0.7 at 600 K.^[^ ^26^ ^]^ Such a result makes the developed Cu_6_Te_3−_ *_x_* S_1+_ *_x_* materials very promising candidates for the harvesting of low parametric waste heat near room temperature or even thermoelectric cooling after further optimization. In contrast to well‐known Cu_2_(S,Se,Te) binaries which show high *zT* values only in the high‐temperature region (above 800 K), the Cu_6_Te_3−_ *_x_* S_1+_ *_x_* materials show their *zT*_max_ at ≈500 K (Figure 5b). Therefore, Cu_6_Te_3−_ *_x_* S_1+_ *_x_* materials may serve as the low‐temperature segment in a Cu‐based chalcogenide thermoelectric leg, working in concert with the high‐temperature Cu_2_ *X* segment.

Although the *zT* parameter is one of the highest reported so far for p‐type Cu‐based chalcogenides in the low‐temperature range, the ultimate applicability of the materials has to be assessed using the device *ZT*.^[^ ^65^ ^]^ Therefore, we carried out this estimation and found that the device *ZT* for the best material Cu_6_Te_2.3_S_1.7_ reached 0.83 at Δ*T = *300 K (Δ*T*  =  *T*_h_‐*T*_c_) (Figure 5c), which is the highest value obtained at any time for the Cu‐based chalcogenides in the temperature range of 298–598 K. The estimated maximum energy conversion efficiency *η_max_* ($\eta_{\max}=\frac{\Delta T}{T_{h}}\frac{\sqrt{1+ZT}-1}{\sqrt{1+ZT}+\frac{T_{c}}{T_{h}}}$), for the TE leg developed using the Cu_6_Te_3−_ *_x_* S_1+_ *_x_* materials, can reach 10.7% (Figure 5d).

**Figure 6:**
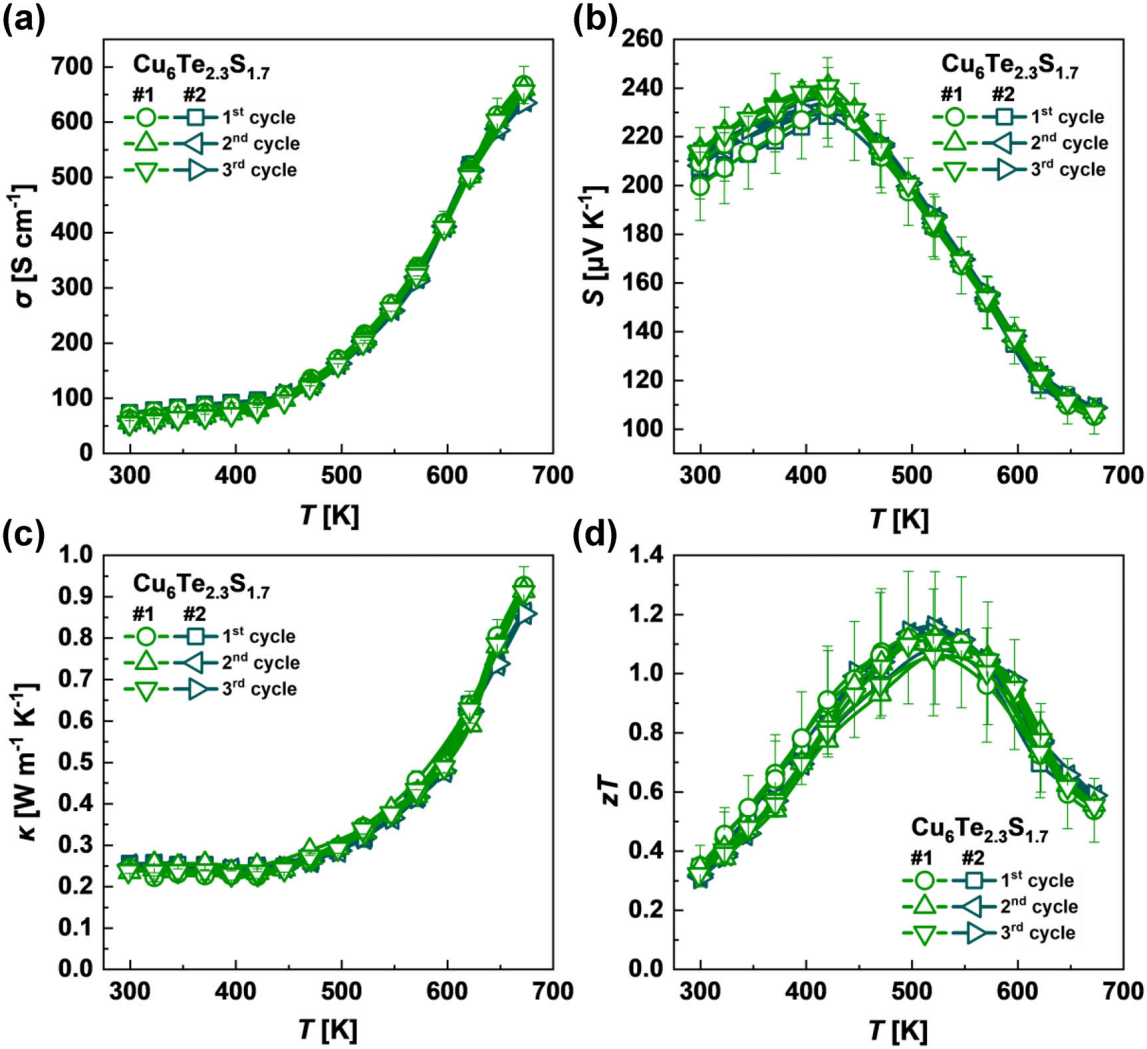
Temperature‐dependent (a) Seebeck coefficient, b) electrical conductivity, c) thermal conductivity, and d) thermoelectric figure of merit *zT* in three heating‐cooling cycles of the best performing Cu_6_Te_2.3_S_1.7_ samples.

In addition to the high thermoelectric figure of merit *zT* parameters of developed Cu_6_Te_3−_ *_x_* S_1+_ *_x_* chalcogenides, the prepared two samples of the best performing Cu_6_Te_2.3_S_1.7_ material show the impressive repeatability and reproducibility of TE properties with only slight deviations within the uncertainly of measured parameters (**Figure** **6**). This is a very rare phenomenon among the Cu‐based chalcogenides, where copper‐ion migration and structural transitions usually lead to the degradation of material and unrepeatable properties, especially in argyrodites,^[^ ^28^, ^29^ ^]^ tetrahedrites^[^ ^18^ ^]^ and Cu‐based binary chalcogenides Cu_2_(S,Se,Te).^[^ ^33^ ^]^ This fact once again underlines the high practical interest in the developed new Cu_6_Te_3−_ *_x_* S_1+_ *_x_* chalcogenides to be used for thermoelectric energy conversion.

## 3 Conclusion

In this work, a remarkable thermoelectric performance has been discovered in new Cu_6_Te_3−_ *_x_* S_1+_ *_x_* chalcogenides. The Cu_6_Te_3_S is characterized by the separate crystal positions of Te and S. The substitution of Te by S in Cu_6_Te_3−_ *_x_* S_1+_ *_x_* not only changes the crystal structure symmetry and creates disorder in the chalcogen sublattice but also eliminates the negative effect of polymorphic phase transition which was reported for Cu_6_Te_3_S. The new γ‐Cu_6_Te_2.3_S_1.7_ polymorph shows a significantly higher Seebeck coefficient of ≈200 µVK^−1^ compared with the very low value of 8.8 µVK^−1^ detected for the Cu_6_Te_3_S material at 298 K. Such an outstanding enhancement in the *S* value was possible due to the optimized carrier concentration *n*_H_ and increased DOS effective mass *m* ^*^. The developed Cu_6_Te_3−_ *_x_* S_1+_ *_x_* materials possess ultralow values of lattice thermal conductivity of ≈0.2–0.25 Wm^−1^K^−1^ at room temperature, which is one of the lowest among crystalline solids and is twice lower than in Cu_2_(S,Se,Te). The ultralow *κ*_L_ is mainly coming from the intensified anharmonicity of lattice vibrations reflected in the high Grüneisen parameters and S/Te substitutional defects.

The synergistic effect of the well‐tuned carrier concentration and the ultralow lattice thermal conductivity leads to a remarkable improvement of the *zT* parameter of Cu_6_Te_3−_ *_x_* S_1+_ *_x_* materials with *x* > 0. In particular, the dimensionless thermoelectric figure of merit *zT* reaches a peak value of 1.12 for the sample with *x = *0.7 at a relatively low temperature of 498 K. However, what is even more important, the developed new Cu‐based chalcogenides show good repeatability and reproducibility of thermoelectric properties on several cycles. The evaluated energy conversion efficiency reaches the excellent value of 10.7 % at a temperature gradient of 375 K (*T*_c = _298 K) for the Cu_6_Te_2.3_S_1.7_ material due to the enhancement of the device *ZT*. This work reveals that the discovered Cu_6_Te_3−_ *_x_* S_1+_ *_x_* materials can become a cheaper and more environmentally friendly alternative to Bi_2_Te_3_‐based materials and can be used to construct thermoelectric converters for waste heat recovery. Moreover, the further carrier concentration tuning may produce the maximum *zT* even at room temperature opening the potential of this material for cooling applications.

## 4 Experimental Section

### Preparation

All samples with the chemical composition Cu_6_Te_3−_ *_x_* S_1+_ *_x_* (0 ≤ *x* ≤ 1) were synthesized in quartz glass ampoules coated with graphite, which were evacuated to a residual pressure of 10^−5^ mbar and sealed with a flame of an oxygen‐gas burner. The ampoules underwent thorough cleaning, which involved washing in a concentrated acid solution with a 1HNO_3_:3HCl ratio, followed by multiple rinses with distilled water and isopropanol before being dried. The synthesis was carried out using high‐purity elements: Cu (99.99%), S (99.999%), and Te (99.999%), all sourced from Alfa Aesar. Precise stoichiometric amounts of the elements were sealed in evacuated quartz ampoules, which were then heated to 1373 K at a rate of 3 K min^−1^ and maintained at this temperature for 10 h to ensure full reaction in the liquid phase. Subsequently, the ampoules were cooled to 873 K and subjected to annealing for 150 h. After homogenization annealing, the furnace was inertially cooled to room temperature.

The resulting ingots were finely ground by hand in an agate mortar to produce powders, which were then densified by spark plasma sintering (SPS). The SPS process was conducted at 723 K for 30 min in a 12.8 mm diameter graphite die, applying axial compressive stress of 45 MPa in an argon atmosphere. During this process, the heating and cooling rates were set to 70 and 20 K min^−1^, respectively. The sintered pellets, ≈2 mm thick, were polished and prepared for measurements of transport properties.

### Characterization

The phase identification was carried out on a BRUKER D8 Advance X‐ray diffractometer with the use of Cu *K*α radiation (*λ*  =  1.5418 Å, Δ2*Ѳ*  =  0.007 °, 2*Ѳ* range 10 – 100 °) with Bragg–Brentano geometry. The WinCSD software package was used to estimate the lattice parameters by least squares refinement.^[^ ^66^ ^]^

Thermal analysis was conducted using a differential scanning calorimeter (NETZSCH DSC 404 F3 Pegasus) on samples of 15 mg in sealed aluminum crucibles. The measurements were carried out at a heating rate of 10 K min^−1^ under a flow of helium.

For the microstructural and chemical analysis, the samples were first embedded in resin, then ground and polished to a fine finish. Prior to examination, the samples were sputtered with a thin layer of conductive graphite. Scanning electron microscopy (ThermoFisher Scientific Scios 2 Scanning Electron Microscope) equipped with standardless energy dispersive X‐ray spectroscopy (EDS) was used to analyze the homogeneity and local chemical composition of the samples.

The NETZSCH SBA 458 Nemesis was used to measure the Seebeck coefficient *S* and electrical conductivity *σ* across the temperature range of 298 – 673 K in an argon flow. The thermal diffusivity *α*_D_ was measured using the NETZSCH LFA 457 *MicroFlash* instrument. The specimens were first spray‐coated with graphite to minimize errors due to material emissivity and laser beam reflection from the glossy surface of the pellets. The thermal conductivity was calculated by applying the following equation: *κ* = *ρC*_p_ *α*_D_, where *ρ* is the density of the pellets from SPS, as determined by the Archimedes method, and *C*_p_ is the specific heat capacity, estimated by utilizing the Dulong–Petit limit. The measurement uncertainties for the Seebeck coefficient and electrical conductivity were 7% and 5%, respectively, while the uncertainty for thermal diffusivity measurements was 3%. The overall combined uncertainty for determining the thermoelectric figure of merit (*zT*) was ≈20%.^[^ ^67^ ^]^ The Hall effect was examined using the four‐probe method, with a constant electric and magnetic field (*H* = 0.9 T) and a current of 100 mA applied through the sample. The uncertainty in the Hall measurements was ≈10%. The sound velocity was measured at 298 K employing the Olympus Epoch 650 ultrasonic flaw detector. The Seebeck coefficient distribution across the polished surface of the specimens was examined using a Scanning Thermoelectric Microprobe (SThM) which exhibits a resolution of 1 µm.

## Conflict of Interest

The authors disclose the following financial interests and personal relationships that may represent potential competing interests: Oleksandr Cherniushok, Taras Parashchuk, and Krzysztof T. Wojciechowski have a pending patent application #P.450136 submitted to The Patent Office of the Republic of Poland.

## Supporting information



Supporting Information

## Data Availability

The data that support the findings of this study are available from the corresponding author upon reasonable request.
