# Supplementary material for: Discovery of a New Cu‐Based Chalcogenide with High zT Near Room Temperature: Low‐Cost Alternative for the Bi2Te3‐Based Thermoelectrics
Source: Adv Mater. 2025 Mar 21;37(18):2420556. doi: 10.1002/adma.202420556 (PMC12051737; doi:10.1002/adma.202420556)
Supplement: Supplementary file 1 — Supporting Information [file ADMA-37-2420556-s001.docx]

***Supporting Information***

**Discovery of a new Cu-based chalcogenide with high *zT* near room temperature: low-cost alternative for the Bi_2_Te_3_-based thermoelectrics**

Oleksandr Cherniushok^1*^, Taras Parashchuk^1^, G. Jeffrey Snyder^2^,
and Krzysztof T. Wojciechowski^1^

^1^Thermoelectric Research Laboratory, Department of Inorganic Chemistry, Faculty of Materials Science and Ceramics, AGH University of Krakow, Mickiewicza Ave. 30, 30-059 Krakow, Poland

^2^Department of Materials Science and Engineering, Northwestern University, Evanston, IL 60208, USA

*^*^E-mail: sashach@agh.edu.pl*


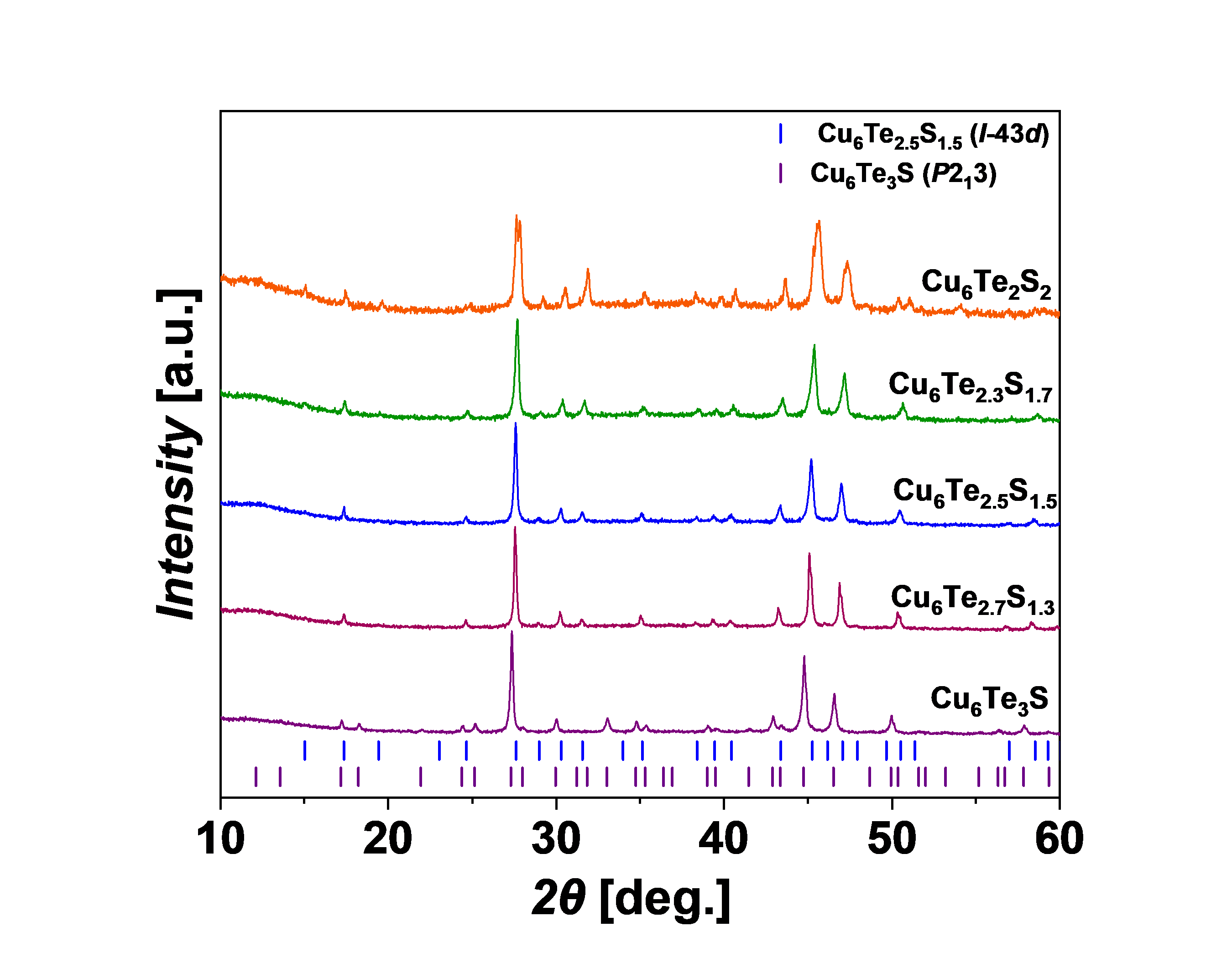


**Figure S1.** Powder X-ray diffraction patterns of the Cu_6_Te_3-_*_x_*S_1+_*_x_* samples pellets after the SPS procedure.

**Figure S2.** EDS elemental map of the polished surfaces of the Cu_6_Te_3-_*_x_*S_1+_*_x_* polycrystalline sample with *x* = 0.7 after LFA measurements.

**Figure S3.** SThM map of Seebeck coefficient on the polished surfaces of the Cu_6_Te_3-_*_x_*S_1+_*_x_* polycrystalline sample with *x* = 0.3 after LFA measurements.

**Figure S4.** The concentration-dependent (a) Seebeck coefficient and (b) Hall mobility for
the Cu_6_Te_3-_*_x_*S_1+_*_x_* samples at 298 K. Points correspond to the experimental measurements, line was calculated using the Kane band model considering the acoustic scattering as the main scattering mechanism.


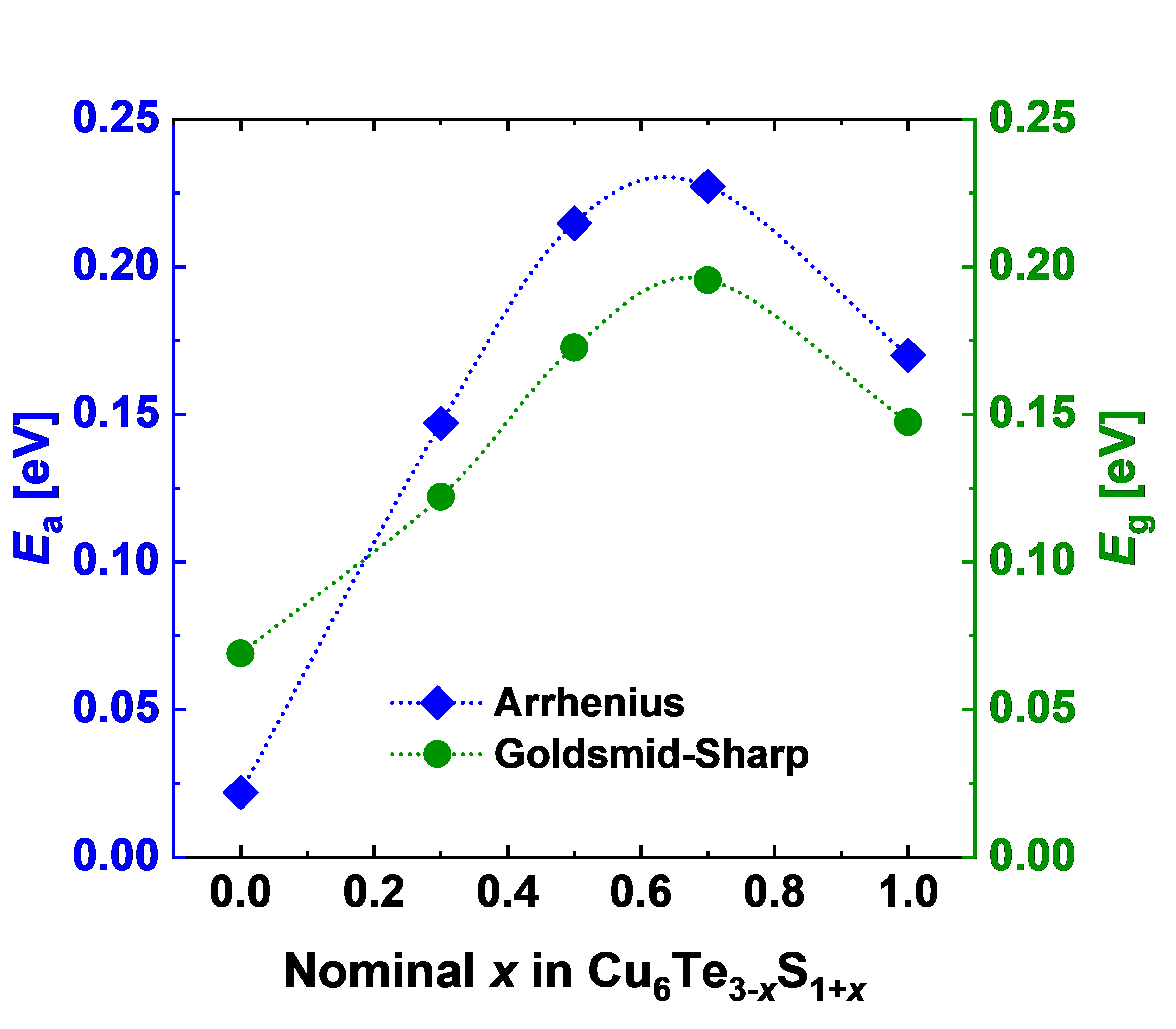


**Figure S5.** The conductivity activation energy *E*_a_ was estimated from the Arrhenius plot of electrical resistivity and the bandgap was estimated from the temperature-dependent Seebeck coefficient using the Goldsmid-Sharp equation for the Cu_6_Te_3-_*_x_*S_1+_*_x_* samples.

**Figure S6.** (a) Temperature-dependent thermal conductivity with subtracted electronic part and (b) calculated Lorenz numbers of Cu_6_Te_3-_*_x_*S_1+_*_x_* materials. (c,d) Estimation of Lorenz number of Cu_6_Te_3_S using isothermal model.

**Details of the Kane band model calculations**

To establish the relationship between the carrier concentration and the Seebeck coefficient we employ the solutions of the Boltzmann transport equations. Considering the Kane band model approximation, the Seebeck coefficient can be found as follows [1,2]:

$S=-\frac{k_{B}}{e}\left[\frac{I_{r+1,2}^{1}\left(\eta,\beta\right)}{I_{r+1,2}^{0}\left(\eta,\beta\right)}-\eta\right]$ (S1)

where *k*_B_, *e*, and *r* denote the Boltzmann constant, charge of an electron, and scattering parameter, respectively; $\eta=\mu/k_{0}T$ and $\beta=k_{B}T/\varepsilon_{g}$ are the reduced chemical potential and the parameter determining the degree of band non-parabolicity, respectively. $I_{n,k}^{m}\left(\eta,\beta\right)$ is two-parametric Fermi integrals:

$I_{n,k}^{m}(\eta,\beta)=\int_{0}^{\infty} \left(-\frac{df}{dx}\right)\frac{x^{m}(x+\beta x^{2})^{n}dx}{(1+2\beta x)^{k}}$ (S2)

The density of electronic states effective mass *m*^*^ which was used for the calculations of the Pisarenko relations was estimated using the experimental Seebeck coefficient *S* and Hall concentration *n* for each sample:

(S3)

**Details of weighted mobility calculations**

Weighted mobility *μ*_w_ was calculated using the following equation [3]:

$\mu_{W}=\frac{3h^{3}}{8\pi e\rho\left(2m_{e}k_{B}T\right)^{3/2}}\left[\frac{\exp\left[\frac{|\alpha|}{k_{B}/e}-2\right]}{1+\exp\left[-5\left(\frac{|\alpha|}{k_{B}/e}-1\right)\right]}+\frac{\frac{3}{\pi^{2}}\frac{|\alpha|}{k_{B}/e}}{1+\exp\left[5\left(\frac{|\alpha|}{k_{B}/e}-1\right)\right]}\right]$ (S4)

**Details of elastic property calculations**

The bulk modulus was calculated using the following equation [4]:

$B=\rho\left(\upsilon_{L}^{2}-\frac{4}{3}\upsilon_{T}^{2}\right)$ (S5)

where *ρ* is the material density.

The shear modulus was calculated as:

$G=\upsilon_{T}^{2}\rho$ (S6)

The Young’s modulus is calculated as:

$E=\frac{9BG}{3B+G}$ (S7)

The Poisson’s ratio is calculated as:

$\nu=\frac{E-2G}{2G}$ (S8)

The Debye temperatures were calculated using the following expression [5]:

$\Theta_{D}=\frac{h}{k_{B}}\left[\frac{3n}{4\pi}\left(\frac{N_{A}\rho}{M}\right)\right]^{1/3}v_{m}$ (S9)

where *h* is Planck’s constant, *N_A_* is Avogadro’s number, *M* is the molecular weight, *n* is the number of atoms in the molecule, and *ν_m_* is the averaged wave velocity integrated over several crystal directions [5]:

$v_{m}=\left[\frac{1}{3}\left(\frac{2}{v_{_{t}}^{3}}+\frac{1}{v_{l}^{3}}\right)\right]^{-1/3}$ (S10)

where *ν_l_* and *ν_t_* are the longitudinal and transverse sound velocities, respectively. Grüneisen parameters *γ* were calculated using the following formula [6]:

$\gamma=\frac{3}{2}\left(\frac{1+\nu}{2-3\nu}\right)$ (S11)

**Details of the thermal transport property calculations**

The phonon mean free paths were calculated by [7]:

$l_{ph}=\frac{3\kappa_{lat}}{C_{V}v_{m}}$ (S12)

Considering the ultrasonic data, the lattice thermal conductivity was also calculated as follows [7]:

$\kappa_{L}=K\frac{\bar{M}\Theta_{D}^{3}\delta}{\gamma^{2}n^{\frac{2}{3}}T}$ (S13)

Here *n* is the number of atoms in the primitive unit cell, *d* ^3^ is the volume per atom, Θ*_D_* is the Debye temperature, $\overline{M}$ is the average mass of the atoms in the crystal, and *K* is a collection of physical constants (*K ≈* 3.1×10^–6^ if *κ*_L_ is in Wm^-1^K^-1^, $\overline{M}$ in amu, and *δ* in Angstroms).

According to Cahill’s formulation based on the maximum phonon scattering approach, the glass limit for the thermal conductivity *κ_glass_* was estimated by:

$\kappa_{glass}=\frac{1}{2}\left(\frac{\pi}{6}\right)^{1/3}k_{B}V^{-2/3}\left(2v_{t}+v_{l}\right)$ (S14)

where *V* is the average volume per atom calculated from the refined lattice parameters.

**References**

[1] Y.I. Ravich, B.A. Efimova, I.A. Smirnov, Semiconducting Lead Chalcogenides, Springer US, 1970. 10.1007/978-1-4684-8607-0.

[2] B.M. Askerov, Electron Transport Phenomena in Semiconductors, 1994. 10.1142/1926.

[3] Snyder, G. J.; Snyder, A. H.; Wood, M.; Gurunathan, R.; Snyder, B. H.; Niu, C. Weighted Mobility. Advanced Materials 2020, 32 (25), 2001537.

[4] E.P. Papadakis, E.P. Papdakis, C.A. Stickels, R.C. Innes, An Ultrasonic Technique for Measuring the Elastic Constants of Small Samples, SAE Trans. 104 (1995) 830–837.

[5] O.L. Anderson, A simplified method for calculating the debye temperature from elastic constants, J. Phys. Chem. Solids. 24 (1963) 909–917. 10.1016/0022-3697(63)90067-2.

[6] D.S. Sanditov, V.N. Belomestnykh, Relation between the parameters of the elasticity theory and averaged bulk modulus of solids, Tech. Phys. 56 (2011) 1619–1623. 10.1134/S106378421111020X.

[7] H. Xie, S. Hao, S. Cai, T.P. Bailey, C. Uher, C. Wolverton, V.P. Dravid, M.G. Kanatzidis, Ultralow thermal conductivity in diamondoid lattices: high thermoelectric performance in chalcopyrite Cu_0.8+y_Ag_0.2_In_1−y_Te_2_, Energy Environ. Sci. 13 (2020) 3693–3705. 10.1039/D0EE02323J.
